# Surgical Procedures Used for Correction of Scheuermann's Kyphosis: A Meta-Analysis

**DOI:** 10.1155/2021/2142964

**Published:** 2021-10-23

**Authors:** Qingshan Li

**Affiliations:** Fourth Department of Orthopedics, Handan Central Hospital of Hebei Province, Handan 056001, China

## Abstract

**Objectives:**

Scheuermann's kyphosis can cause severe back pain and cosmetic disorders to patients. Previous studies on surgical procedure selection for correction of Scheuermann's kyphosis have drawn controversial conclusions. Here, a meta-analysis was performed to figure out a better way between anterior-posterior (AP) combined procedures and posterior-only (PO) procedures.

**Methods:**

We searched PubMed database and Ovid database, as well as Cochrane Library (between January 2009 and December 2020, around recent ten years), for studies reporting Scheuermann's kyphosis correction in an anterior way or a posterior way. Random effects meta-analysis regarding correction degrees and incidence of proximal junctional kyphosis (PJK) was performed.

**Results:**

Finally, 13 unique studies including 586 patients (AP: 300; PO: 286) were identified and included for this meta-analysis. Overall, 6 AP cohorts and 10 PO cohorts were pooled regarding the correction degrees of kyphosis in the analysis, respectively. Pooled correction degrees in AP cohorts were 33.31 (95% CI: 27.48–39.15; *I*^2^ = 86%, *P* < 0.001) and in PO cohorts were 31.16 (95% CI: 26.97–35.35; *I*^2^ = 81.1%, *P* < 0.001). Comparison of correction between AP and PO cohorts did not indicate any significant difference. Likewise, postoperative PJK incidence showed no difference. Back pain can be caused by both AP and PO procedures, but which causes less pain remains to be conclusive. The PO approach showed less blood loss and shorter surgical duration as compared to the AP approach.

**Conclusions:**

In summary, this meta-analysis shows similar treatment effects between AP and PO procedures in correcting Scheuermann's kyphosis, suggesting the advantage of PO procedures due to less blood loss and surgical duration. However, the postoperative complications PJK and distal junctional kyphosis (DJK) cannot be well concluded due to the limitation of existing data.

## 1. Introduction

Scheuermann's kyphosis (SK) is a rigid developmental thoracic kyphosis, which can cause severe back pain and cosmetic disorders to patients [1, 2]. Although conservative treatment measures are initially applied, surgical treatment is indicated for kyphosis that is over 70–75 degrees, with significant pain that has not responded to conservative management, and/or respiratory problems due to severe kyphosis, and neurological issues [3–5]. The surgical treatment consists of two different ways; one is the combined anterior-posterior approach (AP) and the other is a posterior-only way (PO), with various types of anchors [6, 7].

However, previous studies on surgical procedure selection for correction of SK have drawn contradictory conclusions. It was reported that a sufficient correction can be achieved by the PO approach, but the AP approach was more likely to get into a satisfying correction [8]. By contrast, a comparative study reported that the PO approach was more successful with a lower incidence of complications, as compared to the AP approach [9]. Interestingly, Koller et al. [10] found that both approaches achieved similar degrees of correction with higher fusion level in the PO group, after comparing the AP with PO procedures in correcting kyphosis.

Considering the controversy stated above, in this study, a meta-analysis was performed in order to figure out a better way between AP and PO procedures in treating SK patients.

## 2. Materials and Methods

### 2.1. Ethical Statement

The ethical approval was waived because all analyses were based on previously published studies.

### 2.2. Literature Search

We searched PubMed database and Ovid database, as well as Cochrane Library (between January 2009 and December 2020, around recent ten years), for studies reporting SK correction in an anterior way or a posterior way. Articles should be written and published in English. Literature search for studies of interest should include the following terms: (1) Scheuermann's kyphosis AND posterior fusion or (2) Scheuermann's kyphosis AND anterior fusion.

### 2.3. Inclusion Criteria

All included studies should have at least reported the outcome of kyphosis correction, recruiting a cohort of Scheuermann's disease patients who underwent AP surgery or PO surgery, regardless of comparative or noncomparative studies. Here, we focus on studies of surgical procedure selection for correction of SK based on the effect of kyphosis correction.

### 2.4. Data Extraction

First, all related article titles and abstracts were screened and only original research was included. Second, full-length relevant articles were intensively read and checked in detail. At last, baseline information was extracted, as well as the raw data regarding follow-up time, patient age, sex distribution, sample size, Cobb angle, correction degrees, correction rate, blood loss, surgical duration, and postoperative complications including proximal junctional kyphosis (PJK) and distal junctional kyphosis (DJK).

### 2.5. Quality Assessment of Included Studies

All included studies in this meta-analysis were retrospective case-control studies or observational cohort studies. Thus, Newcastle–Ottawa quality assessment scale (9 points) was suitable for quality assessment and used to evaluate the quality of included studies [11].

### 2.6. Measures of Treatment Effect

Both continuous and dichotomous outcomes were generated in this study. Weighted mean difference (WMD) and 95% confidence interval (CI) were generated for continuous outcomes. Also, the odds ratio (OR) and 95% CI were calculated for dichotomous outcomes.

### 2.7. Assessment of Heterogeneity

Distributed as *χ*^2^ statistics, *Q* statistics was used to evaluate heterogeneity, with its *P* values revealed by the forest plot. The heterogeneity test was considered statistically significant when *P* < 0.10. Simultaneously, *I*^2^ was used to estimate the size of the heterogeneity. *I*^2^ > 50% indicated considerable heterogeneity among the included studies, and then a random effects analysis should be performed in meta-analysis.

### 2.8. Test for Risk of Publication Bias

Funnel plot was not performed to determine risk of publication bias due to the small number of included studies. Begg's and Egger's tests were used to assess the publication bias.

### 2.9. Statistical Analysis

All data analyses were conducted with software STATA 12.0 (Stata Corporation, College Station, TX, USA). Random effects meta-analysis regarding correction degrees and incidence of PJK was performed. Heterogeneity was assessed by *I*^2^ statistic. *P* values were set at 0.10 as significant in assessment of heterogeneity, Begg's test, and Egger's test [12, 13]. In the rest of all, *P* < 0.05 was regarded as statistically significant. All *P* values were presented as two-tailed.

## 3. Results

### 3.1. Literature Search

**Figure 1:**
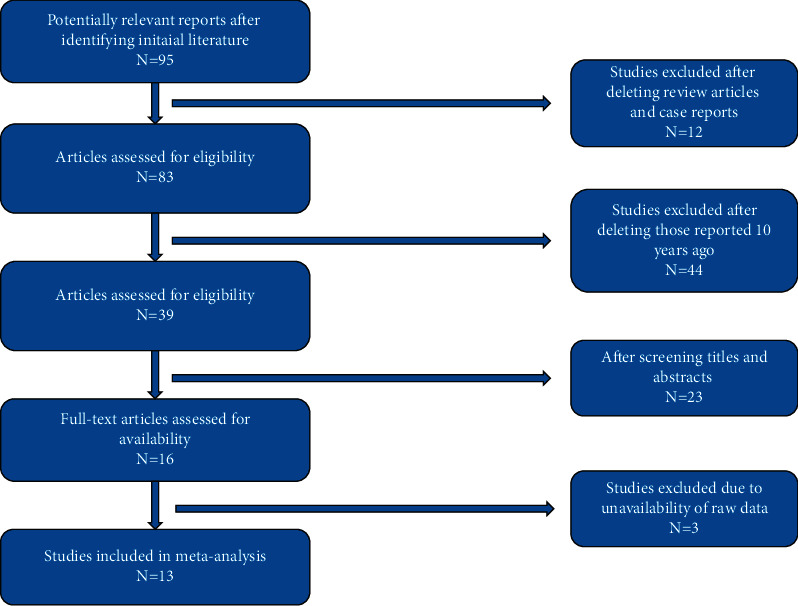
Flow diagram for study selection.

As presented in Figure 1, after database search, there were 95 relevant papers included in the first-round literature selection. After study selection, 13 unique studies [4, 6, 10, 14–22] including 586 patients (AP: 300; PO: 286) were identified and included for this meta-analysis. Overall, 6 AP cohorts and 10 PO cohorts were pooled regarding the correction degrees of kyphosis in the analysis, respectively. Three reports were excluded due to unavailability of raw data [23–25].

### 3.2. Quality Assessment of Included Studies

A summary of quality assessment for each included study is shown in Table 1. Overall, three studies scored 7 points, eight scored 8 points, and two scored 9 points. The methodological quality of all included studies was found to be relatively high.

### 3.3. Characteristics of Included Studies

As shown in Tables 2–4, we extracted baseline information and relevant raw data regarding follow-up time, patient age, sex distribution, sample size, Cobb angle, correction degrees, correction rate, blood loss, surgical duration, and postoperative complications including PJK and DJK. All studies were retrospective in design. Follow-up time ranged from 22.8 months to 216 months. Patient age was between 11 and 44 ± 8 years. Also, most patients were males.

### 3.4. Pooled Analysis of Kyphosis Correction

**Figure 2:**
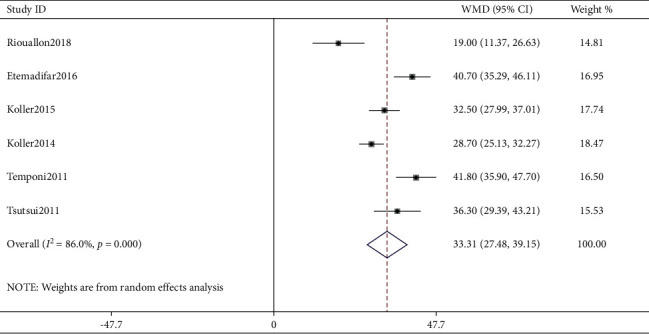
Forest plot of kyphosis correction by the combined anterior-posterior approach.

As shown in Figure 2, six studies [6, 10, 16, 19, 21, 22] reported the correction effect by AP and were pooled into the meta-analysis. As a result, pooled correction degrees in AP cohorts were 33.31 (95% CI: 27.48–39.15; *I*^2^ = 86%, *P* < 0.001). Because the study (Koller et al. [10]) might have recruited in the AP cohort 46 patients that were included in another study (Koller et al. [19]), we have revised the pooled analysis of AP group with the study (Koller et al. [10]) excluded; then the pooled correction degrees in AP cohorts were 33.45 (95% CI: 25.97–40.92; *I*^2^ = 88.8%, *P* < 0.001).

**Figure 3:**
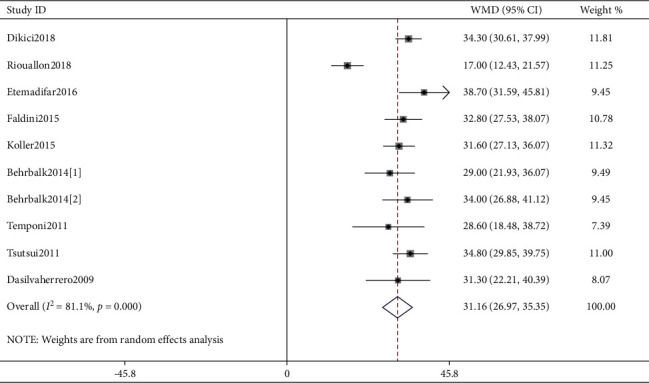
Forest plot of kyphosis correction by the posterior-only approach.

As shown in Figure 3, nine studies [5, 6, 10, 14, 17, 21, 22] reported the correction effect by PO, and one [14] of the included studies reported two PO cohorts. Thus, totally ten PO cohorts were pooled into the meta-analysis. Pooled correction degrees in PO cohorts were 31.16 (95% CI: 26.97–35.35; *I*^2^ = 81.1%, *P* < 0.001).

**Figure 4:**
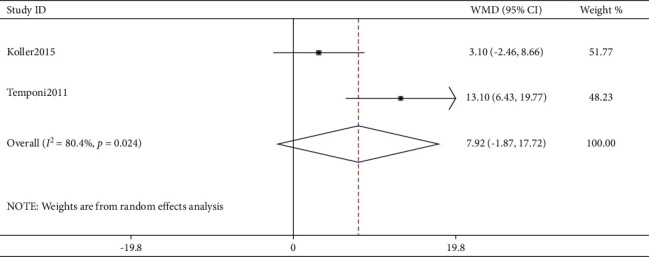
Comparison of kyphosis correction between the combined anterior-posterior approach and the posterior-only approach.

As shown in Figure 4, only two studies [10, 21] compared the correction effect between AP and PO cohorts, and when pooled together for further analysis, the comparison did not indicate any significant difference (*P* > 0.05).

**Figure 5:**
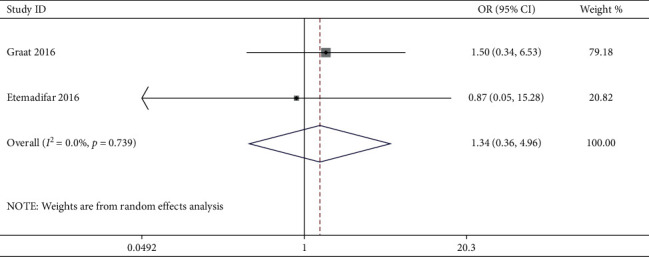
Comparison of proximal junctional kyphosis (PJK) incidence between the combined anterior-posterior approach and the posterior-only approach.

Likewise, only two studies [16, 18] compared postoperative PJK incidence between AP and PO cohorts, and pooled analysis of PJK incidence showed no difference, as shown in Figure 5. Four studies [4, 5, 15, 18] have reported incidence of distal junctional kyphosis (DJK), but no studies compared the incidence of postoperative DJK between AP and PO cohorts. The PJK incidence was reported to range from 0% to 31%. Also, only two studies [16, 20] have reported the surgical data (blood loss and surgical duration), and clearly, the PO approach showed less blood loss and shorter surgical duration as compared to the AP approach.

### 3.5. Assessment of Pain

As some patients with kyphosis deformity suffer from back pain, we here also incorporated the pain assessment based on the available data. Among all studies included, 4 studies have assessed pain status change and recorded as visual analogue scale (VAS) score [6, 18, 20, 21]. Riouallon et al. [6] performed a comparative study including 131 patients, 79% cases undergoing correction surgeries because of severe back pain and 21% due to cosmetic disorders. They followed up 85 patients for more than one year after surgeries and found that most patients (81%) did not suffer postoperative back pain but 19% patients still suffered back pain of different degrees. Graat et al. [18] performed a long-term follow-up of 28 patients postsurgery and compared them regarding back pain; it was found that the AP group suffered less pain than the PO group, while Temponi et al. [21] reported the opposite result to that. Koptan et al. [20] reported that all patients complained of pain preoperatively but did not give further information.

### 3.6. Publication Bias Assessment

**Figure 6:**
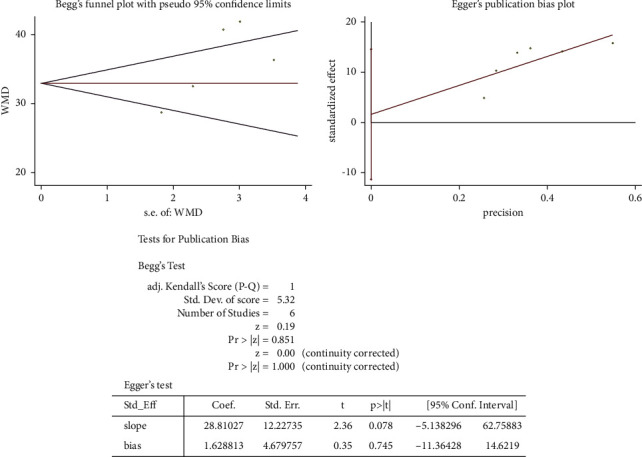
Publication bias analysis by Begg's and Egger's tests regarding the combined anterior-posterior approach.

**Figure 7:**
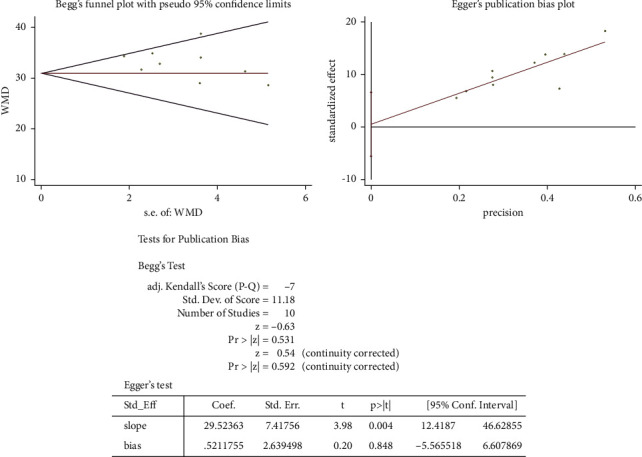
Publication bias analysis by Begg's and Egger's tests regarding the posterior-only approach.

As shown in Figure 6, no publication bias was found relevant to correction of kyphosis in AP cohorts by Begg's rank correlation test and Egger's linear regression test (both *P* > 0.10). Likewise, Figure 7 showed no publication bias with regard to correction of kyphosis in PO cohorts (both *P* > 0.10).

## 4. Discussion

To the best of our knowledge, the PO approach was the first surgical technique introduced to correct SK deformity and was first performed by Bradford in 1975 [4, 26]. Many clinical and radiological results have reported that PO fusion is an efficient technique for the treatment of SK [4, 6, 10, 14, 16, 17, 21]. Different methods have been introduced over the past few years, and combined AP fusion has been recommended more suitable for rigid and major deformities for many years [10, 19], but complication rates, operation time, and blood loss were significantly higher in AP procedures [16]. Nowadays, debates continue regarding surgical strategy selection between AP and PO fusion for the surgical management of SK [27].

In this study, we performed a meta-analysis in an effort to identify a better approach from AP and PO fusion procedures for correcting SK deformity. The research focus was on the correction effect reflected by achieving more correction degrees, and postoperative complications including PJK were also compared between the two groups, although DJK cannot be compared due to the lack of reports. In our study, six studies reported the correction effect by AP and were pooled into the meta-analysis. As a result, pooled correction effect in AP cohorts was 33.31 degrees (WMD, 95% CI: 27.48–39.15). In addition, nine studies reported the correction effect by PO, and one of them reported two PO cohorts. Thus, totally ten PO cohorts were pooled into the meta-analysis. Also, pooled correction effect in PO cohorts was 31.16 degrees (WMD, 95% CI: 26.97–35.35). Comparing the correction effect between the AP approach and the PO approach, there was no significant difference found although only two studies compared AP cohorts to PO cohorts (*P* > 0.05). Likewise, only two studies compared postoperative PJK incidence between AP and PO cohorts, and pooled analysis of PJK incidence showed no difference. Unfortunately, there were no studies having compared the incidence of postoperative DJK between AP and PO cohorts, though four studies have demonstrated DJK incidence ranging from 0% to 31%. Also, only two studies have reported the surgical data (blood loss and surgical duration), and apparently, the PO approach showed less blood loss and shorter surgical duration.

A previous meta-analysis [7] has revealed that the pooled correction loss of Cobb angle for the AP group was 4.1 (95% CI: 3.4–4.8), and for the PO group, it was 3.8 (95% CI: 3.3–4.4), without any significant difference indicated by the results. This report is consistent with our meta-analysis results that there was no difference with regard to the change of Cobb angle before and after surgery between the AP group and the PO group. Moreover, it was reported that the PO group showed advantages in blood loss, surgery time, and junctional kyphosis [7]. It was in line with our results that the PO group showed less blood loss and shorter surgery duration. Our analytical results, however, did not indicate any difference regarding the postoperative PJK incidence due to the lack of raw data that were available. That meta-analysis has included a wide range of studies that were published between 1964 and 2012, and those studies varied too much, especially considering that the surgical techniques are ongoing in progress. To overcome the shortcomings, we only included eligible studies published between 2009 and 2020, within around recent ten years. Seven new published articles [4, 6, 10, 16–18] have been included in our meta-analysis, which is a helpful update to that previous meta-analysis [7]. Recently, another meta-analysis showed that PO surgery and AP surgery achieved comparable treatment effects of SK disease, which is consistent with our results [28]. However, that study goes with the limitations that most of the studies included were published ten years ago, and thus that meta-analysis missed some important up-to-date literature.

As to publication bias assessment in this study, there were no publication bias found relating to correction of kyphosis in AP cohorts by Begg's rank correlation test and Egger's linear regression test (both *P* > 0.10). Likewise, it also showed no publication bias with regard to the correction of kyphosis in PO cohorts (both *P* > 0.10). Thus, this meta-analysis is in a good quality in terms of publication bias.

However, we have to demonstrate some potential limitations that may exist in this work. To start with, only English-written studies were selected and included in this meta-analysis, potentially excluding some relevant reports written in other languages, due to a language limitation. Additionally, the number of patients included in both groups was relatively small (AP: 300 vs. PO: 286), which cannot be neglected in the interpretation of findings in this meta-analysis. At last, all included studies in the pooled analysis were retrospective in design and most are noncomparative studies, thus might reduce the power of this work.

## 5. Conclusions

In summary, this meta-analysis shows similar treatment effects between AP and PO procedures in correcting Scheuermann's kyphosis, suggesting the advantage of PO procedures due to less blood loss and surgical duration. However, the postoperative complications PJK and DJK cannot be well concluded due to the limitation of existing reports.

## Figures and Tables

**Table 1:** Quality assessment of included studies by Newcastle–Ottawa Quality Assessment Scale.

Study	Selection	Comparability	Exposure	Total score
Dikici et al. [5]	3	2	3	8
Riouallon et al. [6]	3	2	3	8
Cobden et al. [4]	2	2	3	7
Graat et al. [18]	3	2	3	8
Etemadifar et al. [16]	4	2	3	9
Faldini et al. [17]	3	2	2	7
Koller et al. [10]	3	2	3	8
Koller et al. [19]	3	2	3	8
Behrbalk et al. [14]	2	2	3	7
Temponi et al. [21]	3	2	3	8
Tsutsui et al. [22]	3	2	3	8
Dasilvaherrero et al. [15]	3	2	3	8
Koptan et al. [20]	4	2	3	9

**Table 2:** Characteristics of included studies.

Study	Country	Study design	Follow-up (months)		Age (yrs)		Sex		No. of patients	
			AP	PO	AP	PO	M	F	AP	PO
Dikici et al. [5]	Turkey	Retrospective	—	36	—	18.6 ± 3.4	20	19	—	39
Riouallon et al. [6]	France	Retrospective	57	57	Overall: 23 ± 10		81	50	64	67
Cobden et al. [4]	Turkey	Retrospective	—	41	—	19 (15–36)	18	2	—	20
Graat et al. [18]	Netherlands	Cohort study	216	216	Overall: 44 ± 8		—	—	16	13
Etemadifar et al. [16]	Iran	Prospective	69.6	45.6	20.9 ± 5.3	19.3 ± 2.7	20	10	16	14
Faldini et al. [17]	Italy	Retrospective	—	25.2	—	19.6 (13–24)	—	—	—	20
Koller et al. [10]	Germany/US	Matched-pair study	—	—	23.6 ± 11.4	20.7 ± 10.4	—	—	46	46
Koller et al. [19]	Germany	Retrospective	24	—	23.6 ± 10.8	—	74	37	111	—
Behrbalk et al. [14]	UK	Retrospective	—	≥24	—	22 ± 8	8	2	—	10
Behrbalk et al. [14]	UK	Retrospective	—	≥24	—	19 ± 6	10	1	—	11
Temponi et al. [21]	Brazil	Case-control	37.5	22.8	19	27.3	22	6	19	9
Tsutsui et al. [22]	US	Retrospective	—	—	15.1 (13–17)	14.8 (11–19)	13	9	11	11
Dasilvaherrero et al. [15]	Brazil	Retrospective	—	65.8 ± 39.92	—	16.8 ± 2.89	7	3	—	10
Koptan et al. [20]	Egypt	Retrospective	≥24	≥24	16 ± 0.7	15 ± 0.6	12	21	17	16

AP, combined anterior-posterior approach; PO, posterior-only approach; OP, operation; M, male; F, female.

**Table 3:** Kyphosis correction of the patients included in all studies.

Study	Cobb angle (pre-op)		Cobb angle (post-op)		Correction (degree)		Correction rate	
	AP	PO	AP	PO	AP	PO	AP	PO
Dikici et al. [5]	—	73.3 ± 7.9	—	39 ± 8.7	—	—	—	46% ± 13
Riouallon et al. [6]	76 ± 23	78 ± 13	57 ± 21	61 ± 14	—	—	—	—
Cobden et al. [4]	—	79.8	—	44.9	—	—	—	—
Graat et al. [18]	85	79	62.1	65.6	—	—	27%	17%
Etemadifar et al. [16]	83.7 ± 8.1	81.9 ± 9.4	43 ± 7.5	43.2 ± 9.8	42.2	41.8	50.5%	51%
Faldini et al. [17]	—	78.6 ± 11.2	—	45.8 ± 4.4	—	—	—	—
Koller et al. [10]	75.9 ± 9.6	78.7 ± 10.1	43.4 ± 12.3	47.1 ± 11.7	33.7 ± 14.7	30.6 ± 12.4	—	—
Koller et al. [19]	67.2 ± 12.2	—	38.5 ± 14.8	—	28.9 ± 13.4	—	—	—
Behrbalk et al. [14] [1]	—	72 ± 7	—	43 ± 9	—	29 ± 9	—	—
Behrbalk et al. [14] [2]	—	78 ± 9	—	44 ± 8	—	34 ± 6	—	—
Temponi et al. [21]	77.6 ± 10.4	72.9 ± 12.0	35.8 ± 8.0	44.3 ± 9.8	41.7 ± 12	28.6 ± 6	53.2 ± 11.9	39.3 ± 7.8
Tsutsui et al. [22]	84.9 ± 10.2	82.7 ± 6.4	48.6 ± 5.7	47.9 ± 5.4	—	—	—	—
Dasilvaherrero et al. [15]	—	78.8 ± 7.59	—	47.5 ± 12.54	—	33.9 ± 9.53	—	43.25% ± 12.56%
Koptan et al. [20]	79.8 (65–98)	85.5 (69–102)	—	—	38.8 (37–45)	45.1 (40–49)	48.7%	52.2%

AP, combined anterior-posterior approach; PO, posterior-only approach; op, operation.

**Table 4:** Other information of the patients in all included studies.

Study	Blood loss (mL)		Surgical duration		PJK (case)		DJK (case)	
	AP	PO	AP	PO	AP	PO	AP	PO
Dikici et al. [5]	—	—	—	—	—	—	—	12 (31%)
Riouallon et al. [6]	—	—	—	—	—	—	—	—
Cobden et al. [4]	—	—	—	—	—	3 (15%)	—	3 (15%)
Graat et al. [18]	—	—	—	—	9	6	0	0
Etemadifar et al. [16]	1380	760	545.3 min	263.5 min	1	1	—	—
Faldini et al. [17]	—	—	—	—	—	—	—	—
Koller et al. [10]	—	—	—	—	—	—	—	—
Koller et al. [19]	—	—	—	—	—	—	—	—
Behrbalk et al. [14]	—	—	—	—	—	—	—	—
Temponi et al. [21]	—	—	—	—	—	—	—	—
Tsutsui et al. [22]	—	—	—	—	—	—	—	—
Dasilvaherrero et al. [15]	—	—	—	—	—	1	—	0
Koptan et al. [20]	910	620	315 min	215 min	—	—	—	—

AP, combined anterior-posterior approach; PO, posterior-only approach; op, operation; PJK, proximal junctional kyphosis; DJK, distal junctional kyphosis.

## Data Availability

The data used in this study are all included within this article and open to all readers.

## Abbreviations

AP: Anterior-posterior
CI: Confidence interval
DJK: Distal junctional kyphosis
OR: Odds ratio
PJK: Proximal junctional kyphosis
PO: Posterior-only
SK: Scheuermann's kyphosis
WMD: Weighted mean difference.
